# “A little bit more looking…listening and feeling” A qualitative interview study exploring advanced clinical practice in primary care and community pharmacy

**DOI:** 10.1007/s11096-021-01353-9

**Published:** 2021-11-22

**Authors:** Elizabeth Mary Seston, Ellen Ingrid Schafheutle, Sarah Caroline Willis

**Affiliations:** 1 Centre for Pharmacy Workforce Studies, Division of Pharmacy & Optometry, School of Health Sciences, Faculty of Biology, Medicine and Health, The University of Manchester, Oxford, M13 9PT UK; 2 Innovation Management and Policy Division, Alliance Manchester Business School, The University of Manchester, Booth Street West, Manchester, M15 6PB UK

**Keywords:** Advanced practise, Clinical skills, Community pharmacy, Pharmacist, Physical examination

## Abstract

*Background* Growing demands on healthcare globally, combined with workforce shortages, have led to greater skill mix in healthcare settings. Pharmacists are increasingly moving into complex areas of practice, a move supported by policy and education/training changes. *Aim* To understand the nature of extended roles for pharmacists practising at an advanced level in primary care and community pharmacy settings, to explore how clinical and physical examination was incorporated into practice and to understand the impact of providing such examination on practice and on patient relationships. *Method* Telephone interviews (*N* = 15) were conducted with a purposive sample of pharmacists using clinical and physical examination in their practice in Great Britain. The sample included primary care pharmacists (*N* = 5), community pharmacists (*N* = 4), pharmacists working across settings (*N* = 5) and one working in another primary care setting**.** Participants were recruited through professional networks, social media and snowballing. *Results* Primary care pharmacists and community pharmacists were utilising clinical and physical examination skills in their practice. Some community pharmacists were operating locally-commissioned services for low acuity conditions. Incorporating such examinations into practice enabled pharmacists to look at the patient holistically and enhanced pharmacist/patient relationships. Barriers to practise included lack of timely sharing of patient data and perceived reluctance on the part of some pharmacists for advanced practice. *Conclusion* With growing opportunities to provide patient-focussed care, it remains to be seen whether pharmacists, both in Great Britain and elsewhere, are able to overcome some of the organisational, structural and cultural barriers to advanced practice that currently exist in community pharmacy.

## Impacts on practise

- While pharmacists have been practising at an advanced level in primary care settings for a number of years, an increasing number of community pharmacists are practising at this level, and the experiences of both groups are vital for understanding how better to embed advanced practice.
- The ability to use enhanced skills such as physical examination and comprehensive clinical history-taking enabled pharmacists in both primary and community settings to take greater responsibility for the patient journey.
- The community pharmacy setting posed a number of limitations for pharmacists, not least the limited ability to practise advanced skills, although changes to the pharmacy contract and education and training are likely to enhance the ability of community pharmacists to perform advance practice.

## Introduction

In recent years, growing global pressure in healthcare has led to increased skill mix in a number of settings, with task shifting and labour substitution enabling the development of advanced clinical roles [1, 2]. Advanced clinical practice enables these roles and involves, “*a high degree of autonomy and complex decision makings”*[3]. Globally, there is considerable variation in the extent of advanced clinical practice in pharmacy, the existence of underpinning advanced practice frameworks and a lack of clarity as to what advanced practice is [4–7]. One feature of an advanced scope of practice is the ability to use a range of clinical skills [8]. One such key skill is physical examination, defined as the “*systematic process of evaluating the body,”* incorporating “*inspection*, *palpation*, *percussion* and *auscultation*” [9].

In Great Britain, pharmacists have practised at an advanced level alongside general practitioners (GPs—family doctors) for a number of years [10, 11]. Until recently, community pharmacists’ ability to practise at an advanced level has largely been limited by community pharmacy contracts which incentivise medicines supply [12]. Nonetheless, the scope of practice of community pharmacists has broadened over the past decade to include publicly-funded Minor Ailments schemes (branded as ‘Pharmacy First’ in Scotland) whereby patients eligible for free prescriptions get free medication after an over-the-counter consultation, New Medicine services and Medicines Use Reviews (MURs) [13–15]. Community pharmacists have also supported patients with chronic conditions and successfully substituted for urgent and emergency healthcare services [16–20]. In 2020, the General Practice Community Pharmacist Consultation Service (GP CPCS) was launched in England, allowing patients with low acuity conditions to be referred directly to community pharmacy [21]. In 2020, Pharmacy First Plus launched in Scotland, for patients with acute common conditions out of scope of Pharmacy First, using pharmacists’ independent prescribing expertise [22].

## Aim

With growing demand for pharmacists to perform increasingly complex roles in a number of settings, and against a backdrop of recent contractual and policy changes, the aim of the study was to understand the nature of extended roles for pharmacists practising at an advanced level in primary care and community pharmacy settings [23, 24]. The objectives of the study were to explore how clinical and physical examination was incorporated into practice and to understand the impact of providing clinical and physical examination on practice and relationships with patients.

## Ethics approval

Ethical approval was obtained from the University of Manchester Research Ethics Committee (Ref ID: 2019-6706-10792).

## Method

A purposive sample of primary care and community pharmacists in Great Britain (England, Scotland and Wales) who were providing clinical and physical examinations in their practice were approached via email to participate. The rationale for including primary care pharmacists and community pharmacists was that advanced practice is at a more developed level in primary care settings and may provide useful learning for advanced practice in community pharmacy. Participants were recruited through professional networks and through social media and snowballing. Telephone interviews were conducted by a postgraduate student (and registered pharmacist), Alison Howard (AH). Participants were advised that the interviewer was a pharmacist. A piloted, semi-structured topic guide was used, including questions about job role, type of activities performed, attitudes to and understanding of clinical/physical examination, embedding of advanced practice and impact of advanced role on relationships with healthcare professionals and patients.

Interviews, which lasted up to 45 min, were recorded with consent and transcribed verbatim. Data saturation was reached for the two groups of pharmacists (primary care and community pharmacists) when no new themes were emerging. Transcripts were imported into NVivo 12 (QSR International) and analysed thematically according to the following steps: familiarisation with the data, generation of initial codes, and the search for, review of, and definition of themes [25]. Analysis was led by one researcher (EMS), who then discussed the coding framework and themes with the co-authors (EIS, SCW).

## Results

### Profile of participants

Fifteen pharmacists were interviewed in 2019. Five worked in general practice, four in community pharmacy, two held joint roles in general practice/community pharmacy, three held joint roles in general practice/another setting, and one pharmacist worked in another type of primary care setting (details removed to maintain anonymity). Ten were practising in England, four in Scotland and one in Wales. All but one interviewee held an independent prescribing qualification: the remaining pharmacist held a supplementary prescribing qualification but there was evidence that they provided advanced practice.

### Areas of practice

In general practice, pharmacists reported seeing patients as same day presentations for a number of acute conditions which were restricted to ear, nose and throat (ENT), respiratory, urinary and abdominal conditions. In general practice, reception staff triaged patients to the pharmacist and the pharmacists then triaged (face-to-face or telephone) patients, referring where required, independently examining, diagnosing, planning treatment and follow-up. Some ran chronic condition clinics (e.g., respiratory, diabetes), adjusting doses and starting treatment when appropriate.

The six pharmacists practising at an advanced level in community pharmacy described how they used clinical examination skills.

I operate a sort of integrated drop-in service which allows me to…put into practice…my clinical assessment skills and my prescribing…ability.” [ID10: community pharmacist]

Types of physical examinations performed included examinations for ENT symptoms, eye problems and respiratory conditions. Common areas of practice in community pharmacy included “worried well’ patients with acute conditions, such as sinusitis, tonsillitis, exacerbations of asthma, some respiratory conditions, urinary tract infections (UTIs) and skin presentations. Whilst community pharmacy has traditionally been first port of call for some of these low acuity conditions, such as eye problems, previously conditions such as tonsillitis would necessitate a visit to the GP. It should also be noted that there were restrictions as to the types of infections community pharmacists were able to treat, for example, under the Scottish pharmacy schemes, community pharmacists could only treat uncomplicated lower UTIs in adult females under the age of 65 years.

A number of community pharmacists were operating locally-commissioned services to provide treatment for patients using their independent prescribing skills, with one pharmacist describing this as a “*really expandable*” scope of practice. In some cases, patients with acute minor conditions could be directly referred from a local general practice, with the patient treated under one of the pharmacy-based schemes (e.g. Minor Ailments, Pharmacist First) and if none of these schemes were suitable the pharmacist would independently prescribe.

if it’s out of the scope of the Minor Ailments scheme, we would then look at Pharmacy First…If it’s then out of that …[if]…there’s no red flag symptoms…I can prescribe for them. Patients are referred up here from the practice, so if there’s no same day appointments…they… make an appointment … to be seen.” [ID15: community pharmacist]

### Incorporating clinical and physical examination into practice

Interviewees were asked how they incorporated clinical and physical examination into their practice. In primary care/ general practice and community pharmacy, there was general agreement on the importance of history taking as part of the clinical examination process, with physical examination used to confirm or refute a diagnosis suggested by the history.

I think you get 90% of what you need from the history…the examination…should just confirm or refute your diagnosis from the history.” [ID06: general practice pharmacist]

A number of interviewees described how they structured consultations, noting the importance of a full history and elimination of red flags.

I use all those techniques [inspection, palpation, percussion, auscultation]…[I] start…with the full history taking…eliminating red flags…examining the patient just to make sure that there isn’t anything sinister going on.” [ID05: pharmacist in general practice and community]

Incorporating clinical and physical examination into their practice enabled pharmacists to look at the patient in a more holistic manner, going beyond history-taking and clinical examination to explore patient concerns.

Very much thinking more holistically about what else is going on with this patient because it’s not just about what I’m seeing here and what I’m picking up on examination, it’s what else is making this patient feel the way they’re feeling.” [ID11: general practice pharmacist]

In community pharmacy, clinical and physical examination was regarded as a “*slight expansion*” of existing minor ailment work, albeit limited by what was possible in the setting.

My practice…includes the ability to do a little bit more looking, a little bit more listening and a little bit more feeling to clarify what I’m dealing with…the physical examination and the…clinical investigation that I do…are very much linked and, and specifically tied to certain clinical areas that I am trained to manage…[it] is naturally quite restrictive, for example I do not take blood or do tests” [ID10: community pharmacist]

In acknowledging these limitations, the pharmacist felt that their job was not to treat all patients who came into the pharmacy, but to triage those who did. The ability to clinically examine patients enabled the pharmacist to treat a broader range of patients.

It’s not my job to manage absolutely everything that walks through my door, it’s my job to filter it…there’s now an extra group of patients that I can manage… and that’s a positive thing… being able to refer and advise patients in a more effective way… [it] feels like the natural boundary of what we should be doing in a community pharmacy setting.” [ID10: community pharmacist]

The need for opportunities to regularly practise physical examination skills was highlighted. Regular exposure was regarded as being crucial for developing competence and learning how to differentiate between ‘normal’ and ‘abnormal’.

it’s about that appreciation that you’ve got to go away and practise…you’ve got to hear a lot of normal chests before you know what an abnormal chest sounds like.” [ID09: Community pharmacist]

Those working in the community setting noted the lack of opportunities for practising certain examinations, which meant they did not always believe they could maintain sufficient levels of competence to undertake these types of clinical examinations.

It would be extremely rare when I have used the stethoscope…I don’t think I could maintain the competence with the number of people we see to use it effectively.” [ID03: community pharmacist]

### Embedding advanced practice

Pharmacists practising in primary care were asked how they embedded advanced practice. The organisational culture within the GP practice could act as either a facilitator or barrier to a pharmacist’s integration, with GP support important. Some pharmacists believed that patients lacked an awareness of pharmacists’ skills and abilities and often needed the endorsement of the GP in order for the patient to be willing to see the pharmacist.

When the doctor sat with the patient and said…’I’m booking you in with [pharmacist]…they believed them…it took the highest level of authority in the practice for the patient to believe I was competent.” [ID04: general practice pharmacist]

Whilst patients were unused to community pharmacists performing physical examinations, interviewees reported that patients were receptive to this type of consultation, a key benefit being the perception that patients were less likely to subsequently present to a GP.

[Patients are] really, really chuffed that you’re doing that…you do a top to toe exam, you say ‘it’s a viral infection you’ll get better in 7 days,’ the reassurance…then they’re actually much more likely to do the self-care stuff I find and not present at GP practice.” [ID09: community pharmacist]

In community pharmacy, several factors were identified as enabling the embedding of advanced practice. A team with appropriate skill mix, for example, an accuracy checker to relieve the pharmacist from technical tasks and free them for clinical tasks, enabled advanced practice. The lack of access to patients’ medical notes was cited as a barrier, with the ability to view the patient’s medical history, add contemporaneously to the record and share information with GPs seen as vital for integrated care. Without such read–write access, there were concerns that information may not get to the GP in a timely manner.

If I had direct access to a patient’s record it would take…a fraction of the time…it would also make the information totally live…if a patient I see rocks up at out of hours later in the day then…[the health professional is] going to see my notes… At the moment it’s going to be sat on a GP receptionist desk waiting to be scanned.” [ID10: community pharmacist]

Certain barriers to advanced practice roles were specific to community pharmacy and related to what one pharmacist described as “*natural internal workplace pressures*”. A low dispensing volume permitted this pharmacist more time for consultations, and skill mix was also noted again.

It very much depends on your set up and the team…. whether you can take on new roles… I’m quite fortunate in the pharmacy where I work…we’re very, very low on dispensing volume.” [ID10: community pharmacist].

Some interviewees also described cautious and reactive attitudes of many pharmacists as a potential barrier, as illustrated by the following quote.

Pharmacist are very cautious people generally… sometimes we create too many barriers… have a robot mentality, we just go by the guidelines…but sometimes you have to be more proactive.” [ID12: general practice pharmacist]

### What makes advanced practice work?

Pharmacists working in all settings discussed the importance of building long-term relationships with patients. Those who had moved to primary care from community pharmacy described how a change in mind-set was needed, in order to understand the longer-term nature of the relationship with GP practice patients. This continuity meant that pharmacists were more comfortable making clinical judgements, as they would be able to see the outcome of their decision:

[In] a community pharmacy setting…you’re trying to fix that patient before they leave… it takes a little bit of time and experience to realise that in general practice they’re never really lost, they’re always your patient… you are able to let go and know that you can confidently follow up patients in a safe and effective manner.” [ID02: general practice pharmacist]

…I have a lot more confidence if I stop something, I can see that person again… … I think patients appreciate…continuity.” [ID06: advanced clinical practitioner in general practice]

In community pharmacy, some pharmacists described how advanced practice had resulted in a change in expectations from their customers (patients), as they were expecting more from a consultation. This pharmacist also reflected on the way that it had changed how they approached consultations in the pharmacy and enhanced relationships with patients.

It has changed the way that patients see me… you become…part of the…fabric of their ‘go to’ people… that can be a challenge in terms of workload, but it’s also an opportunity because you get to have really useful conversations with people that you just wouldn’t do if you were chatting to them over the counter.” [ID10: community pharmacist]

Several community pharmacists providing services under Scottish pilot schemes to divert patients from GP practices reported that patients, once they had experienced the service, said they would prefer to choose pharmacy as the first port of call the next time they needed treatment, due to ease of access.

Most patients…say ‘that was amazing, can I just come up here next time? …the verbal feedback is amazing.” [ID15: community pharmacist and general practice pharmacist]

The importance of patient confidence in the practitioner and the significance of independent prescribing was highlighted by general practice pharmacists, who described the positive impact being able to prescribe had on relationships with patients.

Before [Independent prescribing]… if I wanted to change any medication I had to wait for a doctor to sign it…the patient…would sort of start to feel a bit suspicious –‘[they’re] asking me to increase the dose and they can’t sign a prescription’…whereas now I can…I can see the difference definitely in the patient’s perception... [if] you have the authority to do the prescription they…trust you more.” [ID12: general practice pharmacist]

A greater understanding of the patient perspective could lead to an improvement in relations between pharmacist and patient.

It’s made me realise…how important it is to understand things from a patient perspective…I would definitely say my relationship with the patient is a lot better.” [ID05: general practice pharmacist and community pharmacist]

## Discussion

This qualitative interview-based study exploring the role of clinical and physical examination in advanced practice in three countries in Great Britain adds to a small body of evidence on pharmacists’ experiences of advanced clinical practice [10, 11, 26]. Pharmacists in this study took greater account of patient concerns, in accordance with the ICE model of consultation [27]. History-taking played a central role in diagnosis, in line with previous research, which has suggested that history-taking is an essential element of any professional/patient encounter and assists healthcare professionals to come to a diagnosis in about three quarters of consultations [19, 27, 28].

This study explored the qualitative experiences of pharmacists providing advanced practice in primary care and community pharmacy and their insights are valuable in understanding how to advance the wider adoption of advanced practice. The study was conducted with pharmacists drawn from the three countries of Great Britain, albeit pharmacists from Wales were under-represented. As with all studies requiring time for an interview, there is a risk of self-selection bias. Whilst many of the issues experienced by the pharmacists will resonate with pharmacists in other countries, some issues may be specific to pharmacists in Great Britain.

The importance of being able to practise examination skills was highlighted by pharmacists in different settings, with the particular difficulty of getting sufficient experience in examination skills in community pharmacy noted. Normalization Process Theory, used to understand how interventions are implemented, embedded and sustained, may be helpful here to understand why this is so [29]. One aspect of NPT called ‘interactional workability,’ describes whether participants can easily integrate the intervention (for example, physical examination skills) into their existing work. It may be that the community pharmacy setting limits the ability of community pharmacists to practise such skills. Research has previously suggested that is not sufficient for pharmacists to have the capabilities and motivation for advanced practice, but they also need to have the organisational support and opportunities for this, as well as contractual incentives [12, 30, 31].

If community pharmacists are to perform expanded roles, it will be vital that they have sufficient opportunities to practise the skills underpinning their enhanced practice and a supportive environment in which to do so. In a recent commentary on advanced practice, the authors argue that “*exposure to experiential and inter-professional learning at all levels, including clinical situations… is the key to development of pharmacists as clinicians*”[7]. Changes to the education and training of pharmacists, in particular a move to more cross-sector working and improved contractual frameworks, such as the new English Community Pharmacy Contractual Framework (CPCF), which focuses on community pharmacy becoming a more clinically focussed service, may help to facilitate this [23, 24]

All but one pharmacist in this study held an independent prescribing qualification and the majority were using their skills in their advanced practice. Several general practice pharmacists described the key role their prescribing qualification played in this, arguing that it instilled patients with confidence and enhances trust in the pharmacist [32, 33]. Patient-perceived pharmacist expertise has previously been identified as a predictor of the quality of the relationship between pharmacist/ patient, which in turn predicts relationship commitment, and a positive patient/pharmacist relationship has been associated with the efficacy of self-care for patients with diabetes [34, 35].

In parts of Great Britain there has been a move to redirect patients with low acuity “*clinically divertible*” conditions to other healthcare providers, including community pharmacies [36]. Such schemes include the GP CPCS in England, where pharmacists utilise extended skills in clinical and physical examination, and NHS Pharmacy First Plus in Scotland, which allows community-pharmacy based independent prescribers to treat patients with acute conditions [21, 22]. Such services present a real opportunity for community pharmacists to provide advanced clinical practice, acting autonomously, taking responsibility for the entire patient journey for patients with acute conditions and, where relevant and available, utilise their independent prescribing skills in an area where they appear comfortable and where both patients and GPs recognise their skills.

Some interviewees suggested that cultural change was needed, with pharmacists’ attitudes a potential barrier, echoing previous research suggesting that pharmacists lack confidence in physical examination and in their decision-making [37, 38]. Pharmacists’ lack of confidence, fear of new experiences, paralysis when decision-making in ambiguous situations and aversion to risk have previously been identified as key barriers to practice change [39]. Dealing with uncertainty is a fundamental part of making a diagnosis and it could be argued that this is an important skill to be incorporated into future pharmacist training [40]. It is hoped that new standards for the initial education and training of pharmacists, recently approved in Great Britain, will better prepare graduates to work as patient-facing, clinical practitioners [24].

If community pharmacists are to effectively provide integrated care, further barriers may need to be overcome. The importance of fostering positive interpersonal relationships with other healthcare professionals has been recognised and in this study general practice pharmacists noted the importance of GP support/endorsement [41, 42]. Co-location of pharmacists with GPs has been shown to improve collaborative working relationships [43]. Developing such collaborative relationships is likely to be more challenging for community pharmacists, who tend to work in isolation with fewer opportunities for contact with doctors [42]. It may be that community pharmacists providing advanced clinical practice need some form of clinical support, which, though stopping short of full clinical supervision, allows them to become an integrated part of the healthcare team. The lack of access to contemporaneous medical notes is also likely to be a significant barrier. Stakeholders in a recent study all stressed the importance of having “*an appropriate system to share relevant information, allowing pharmacists and GPs two-way flow*” [44]. Although data sharing is noted in the service specifications of the GP CPCS and NHS Pharmacy First, it is not clear if these systems will allow a free flow of data. With a growing move towards integrated care, the ability to share data will be key and is a vital patient safety issue, especially if pharmacists are prescribing medication for patients.

Pharmacists discussed the importance of continuity of care, in line with previous research, [45]. For those moving from community to primary care, this necessitated a change of approach, moving from the traditional model of transactional encounters with patients, to recognising the continuity of the patient relationship [46]. Community pharmacists in the study talked positively about patients returning to the community pharmacy having experienced enhanced services, echoing previous research indicating that such services can improve customer loyalty [47]. Factors associated with acceptance/integration of community pharmacy services include a clear service specification, existing good relationship with the pharmacist and the ability to get the problem dealt with in one session [44].

Changing patient behaviour to divert away from general practice/emergency care is driving the introduction of community-based schemes for patients with low acuity conditions [21, 22]. Community pharmacists in this study reported changes to behaviour, with patients actively choosing to visit the pharmacy. Patient acceptability is likely to be a key factor in the success of schemes that aim to divert patients to pharmacy and public lack of awareness of clinical roles could limit the impact of such services [48]. Regional differences in the commissioning of extended services may impact on patient awareness and pharmacists’ views about embedding advanced practice.

This study took place in 2019 and while the themes are still pertinent, significant changes are taking place, with a move to pharmacists becoming ‘prescribing-ready’ upon registration. It is hoped that the advanced practice described in the study will become routine across pharmacy settings. As advanced practice extends further into community settings both in Great Britain and elsewhere, further research will be required to determine the impact of such practice, including the acceptability to patients and other healthcare professionals.

## Conclusion

Pharmacists are now providing advanced care to their patients in a number of settings, and with a move away from the transactional nature of community pharmacy, there are growing opportunities to provide patient-focussed care. It remains to be seen if organisational, structural and cultural barriers to change can be overcome.
